# The classification algorithms to support the management of the patient with femur fracture

**DOI:** 10.1186/s12874-024-02276-5

**Published:** 2024-07-16

**Authors:** Arianna Scala, Teresa Angela Trunfio, Giovanni Improta

**Affiliations:** 1 Department of Public Health, University of Naples “Federico II”, Naples, Italy; 2 Department of Advanced Biomedical Sciences, University of Naples “Federico II”, Naples, Italy; 3 Interdepartmental Research Center on Management and Innovation in Healthcare, University of Naples “Federico II”, Naples, Italy

**Keywords:** Modelling, Machine learning, Classification algorithms, Femur fracture, Biomedical data analysis

## Abstract

Effectiveness in health care is a specific characteristic of each intervention and outcome evaluated. Especially with regard to surgical interventions, organization, structure and processes play a key role in determining this parameter. In addition, health care services by definition operate in a context of limited resources, so rationalization of service organization becomes the primary goal for health care management. This aspect becomes even more relevant for those surgical services for which there are high volumes. Therefore, in order to support and optimize the management of patients undergoing surgical procedures, the data analysis could play a significant role. To this end, in this study used different classification algorithms for characterizing the process of patients undergoing surgery for a femoral neck fracture. The models showed significant accuracy with values of 81%, and parameters such as Anaemia and Gender proved to be determined risk factors for the patient’s length of stay. The predictive power of the implemented model is assessed and discussed in view of its capability to support the management and optimisation of the hospitalisation process for femoral neck fracture, and is compared with different model in order to identify the most promising algorithms. In the end, the support of artificial intelligence algorithms laying the basis for building more accurate decision-support tools for healthcare practitioners.

## Background

Femoral neck fractures, which are more frequent in elderly patients, are mostly caused by chronic bone diseases (e.g. age-related osteoporosis) and are linked to trauma generally of low energy (accidental falls in a domestic environment), more frequently in women [1] who combine severe osteoporosis with internal pathologies and motor coordination deficits [2]. Total hip replacement is among the most frequently performed procedures in the orthopedic field, in which hybrid or cemented fixation is performed. The use of bone cement can experience a number of complications due to severe symptoms during surgery such as hypoxemia, hypotension and unexpected loss of consciousness, especially for procedures performed in emergencies [3]. Femur fracture in patients over 65 years of age is an important and growing social and health problem associated with an increased risk of mortality and deterioration of general condition due to the development of complications. In fact, the development of complications, most frequently of a cardiological and pulmonary nature, leads to a prolongation of the length of hospital stay (LOS) and often to a rapid return to hospital. Recent studies indicate that in Italy the overall costs (including hospitalisation, rehabilitation, disability pensions and indirect costs) of femoral neck fractures in patients over 65 years of age amount to more than € 1,000 million per year [4, 5]. In recent years, healthcare has undergone significant economic cutbacks, which in many cases have led to a reduction in the number of available beds. A method to quantify the optimal use of beds is the measurement of LOS. The average LOS in the orthopaedic ward is generally longer than 10 days [6] and is greatly correlated with comorbidities. Comorbidities have significant influence on the cost of hospitalization and LOS. Hypertension, deficiency anaemia and fluid and electrolyte disturbances are the most common comorbidities [7]. Costs and LOS as well as the risk of mortality and disability are significantly reduced if surgery is timely. In particular, the Italian guidelines [8] require fractures to be treated no later than 48 h. That’s why the “San Giovanni di Dio and Ruggi d’Aragona” university hospital in Salerno implemented a new Diagnostic Therapeutic Assistance Pathway (DTAP). Scala et al. demonstrated, by applying Lean Six Sigma methodology, that the introduction of this DTAP significantly reduced the duration of pre-operative LOS [9]. DTAPs are used to improve the quality and efficiency of care, reduce variability in care and ensure appropriate care for the greatest number of patients [10]. Previous studies have also shown that their implementation, also combined with management approaches such as Lean and Six Sigma, reduces variability in clinical practice and improves outcomes [11–17].

Mathematical modelling has been used in healthcare for various purposes: Pivonka et al. [18] propose the use of mathematical models for hypothesis testing and assistance in clinical treatment for bone fracture recovery, while Marco et al. [19] study different fracture modelling techniques with the aim of predicting a realistic femur fracture pathway, and finally Tastan et al. [20] evaluate different functional relationships between femur density, length and width as predictors of fracture risk using analytical models and numerical tests.

The use of classification algorithms is not limited to the prediction of diseases or the identification of the correct diagnosis [21, 22] but can help healthcare management in different aspects, such as failure management or environment assessment [23]. With respect to the latter part, there are several applications that use LOS to study patient flow. Harper [24] for example investigated the performance of different types of algorithms on different healthcare datasets to identify the best algorithm. These included predicting LOS by determining that there is no one type that is universally valid. Al Taleb et al. [25], on the other hand, use this approach for early prediction of LOS of stroke patients obtaining results in terms of accuracy very similar to those obtained in this study. Several examples are also available in the orthopedic field. Ramkuram et al. [26] implement ML algorithms to predict LOS and payment in patients undergoing primary total hip arthroplasty, while Han et al. [27] apply them to total knee arthroplasty.

In particular, multiple linear regression (MLR) was used to assess which factors influence LOS in Dengue haemorrhagic fever patients [28] and to evaluate the clinical factors that most influence LOS in adult patients with peritonsillar abscess [29]. Furthermore, Trunfio et al. used regression to predict the LOS of patients with femoral neck fracture at University Hospital of Salerno [30]. In addition to mathematical models, machine learning (ML) models also offer the possibility of predicting patient outcomes. Geneve et al., using ML models, were able to make predictions at the time of hospital admission using information from electronic medical records [31]. Daghistani et al. developed a machine learning-based model to predict LOS for heart patients at the King Abdulaziz Cardiac Center in Saudi Arabia [32]. ML models were also used to predict costs and LOS in days of patients undergoing total hip [33, 34] and knee arthroplasty [35].

In this work, we investigate some of the factors that contribute to increased LOS in treatment of patients with femur fractures. Gender, age, and waiting time before surgery are taken into account as independent variables in the model building. Particular attention is also given to additional specific patients’ characteristics, i.e. the presence of different types of comorbidities. Different machine learning classification algorithms are used and compared with each other in terms of accuracy and predictability, and the potential impact of the studied predictive tools is discussed in the view of their perspective development and use.

The article is structured in paragraphs. In the following one, the Materials and Methods will be presented, specifying the context, the data collection and the selected tools for conducting the analysis, then the results obtained will be presented, which will be extensively commented and discussed, highlighting their strengths and weaknesses, in the last paragraphs.

## Materials and methods

The context is that of “San Giovanni di Dio e Ruggi d’Aragona” University Hospital of Salerno (Italy), which consists of 5 plexes spanning the territory around the metropolitan area of Salerno. It has a total of 1′047 beds, more than 3′000 employees and is organized into 10 Care Departments. The main access point is the Emergency Room for which over 130′000 accesses are recorded annually, of which 18′000 end in hospitalization. In addition to the hospital function, the mission is oriented toward both a teaching function with numerous active courses and clinical research aimed at the development of innovative procedures and pathways.

These aspects are also confirmed in our specific application case. In fact, the hospital has shown itself to be at the vanguard in the creation of specific pathways to ensure that patients with femur fracture have prompt intervention and quick recovery, as demonstrated in the literature [9, 30]. Starting precisely from the relevant literature, we decided to expand the analysis by using additional classification algorithms that would also help us determine risk factors for prolonged hospitalization.

**Fig. 1:**
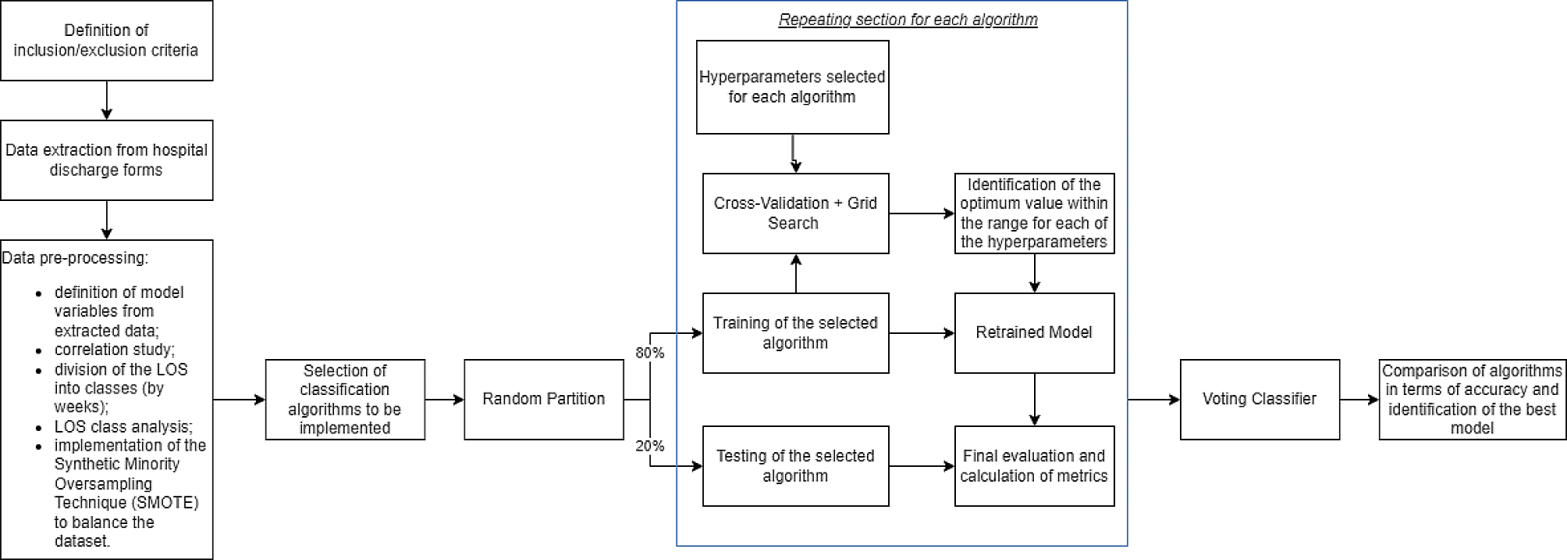
Operative steps followed within the manuscript that led from data collection to results

The workflow followed in this manuscript is shown in Fig. 1.

Google Colaboratory (Colab) As A Service (PAAS) platform provided by Google with one single core hyperthreaded Xeon Processor @2.2 Ghz, 12 GB of RAM and a Tesla T4 GPU, was chosen as a platform to perform the classification analysis since it is an open-source tool that enables advanced data analysis already used in different biomedical studies [36, 37] through the most widely adopted programming language, Python. The main libraries used are io, for importing data from the extraction Excel file, Pandas, NumPy, SMOTE, imported from imblearn.over_sampling, Pyplot for the graphical representations, train_test_split, for the initial partition, Sklearn, for the implementation and study of the ML algorithms.

### Data collection

Data on a sample of 342 patients who underwent femur fracture surgery in 2005–2021 were extracted from the hospital information system. In accordance with the inclusion/exclusion criteria suggested by the national guidelines on essential levels of care, patients with polytrauma or cancer in primary or secondary diagnosis were excluded.

**Fig. 2:**
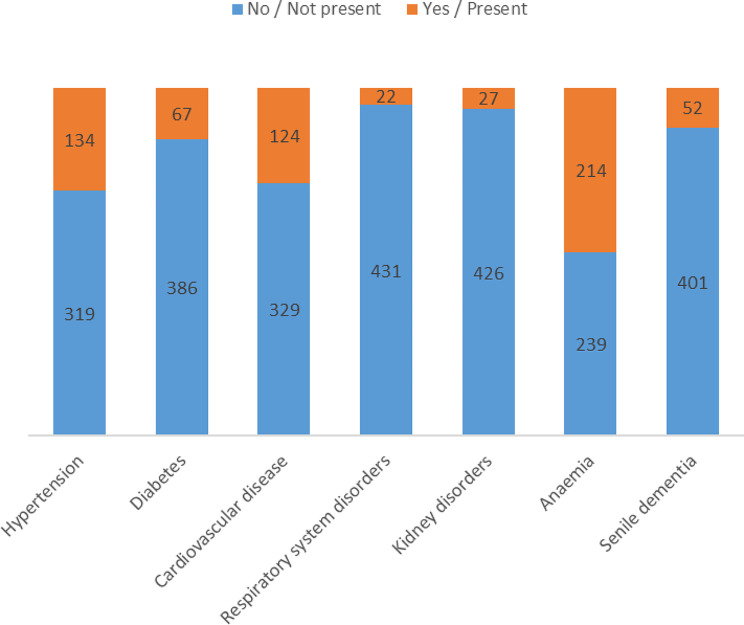
Distribution of the presence of comorbidities in the dataset

The following variables were extracted and taken into account for subsequent analyses:

- Demographic information:
  - Gender;
  - Age;
- Timing information:
  - Year of Discharge;
  - Date and time of admission;
  - Date and time of surgery;
  - Date and time of discharge;
- Festivity:
  - Yes: if the date of admission is a Saturday, Sunday or an Italian national/religious holiday;
  - No: Otherwise;
- Comorbid conditions:
  - Hypertension (Yes/No);
  - Diabetes (Yes/No);
  - Cardiovascular disease (Yes/No);
  - Respiratory disease (Yes/No);
  - Kidney disease (Yes/No);
  - Anaemia (Yes/No);
  - Dementia (Yes/No);

Regarding the selected comorbidities, reference was made to the conditions provided for within the national guidelines to be searched for within the hospitalization grouped together for simplicity in training the model. A graph showing their distribution in the dataset is shown in Fig. 2.

**Fig. 3:**
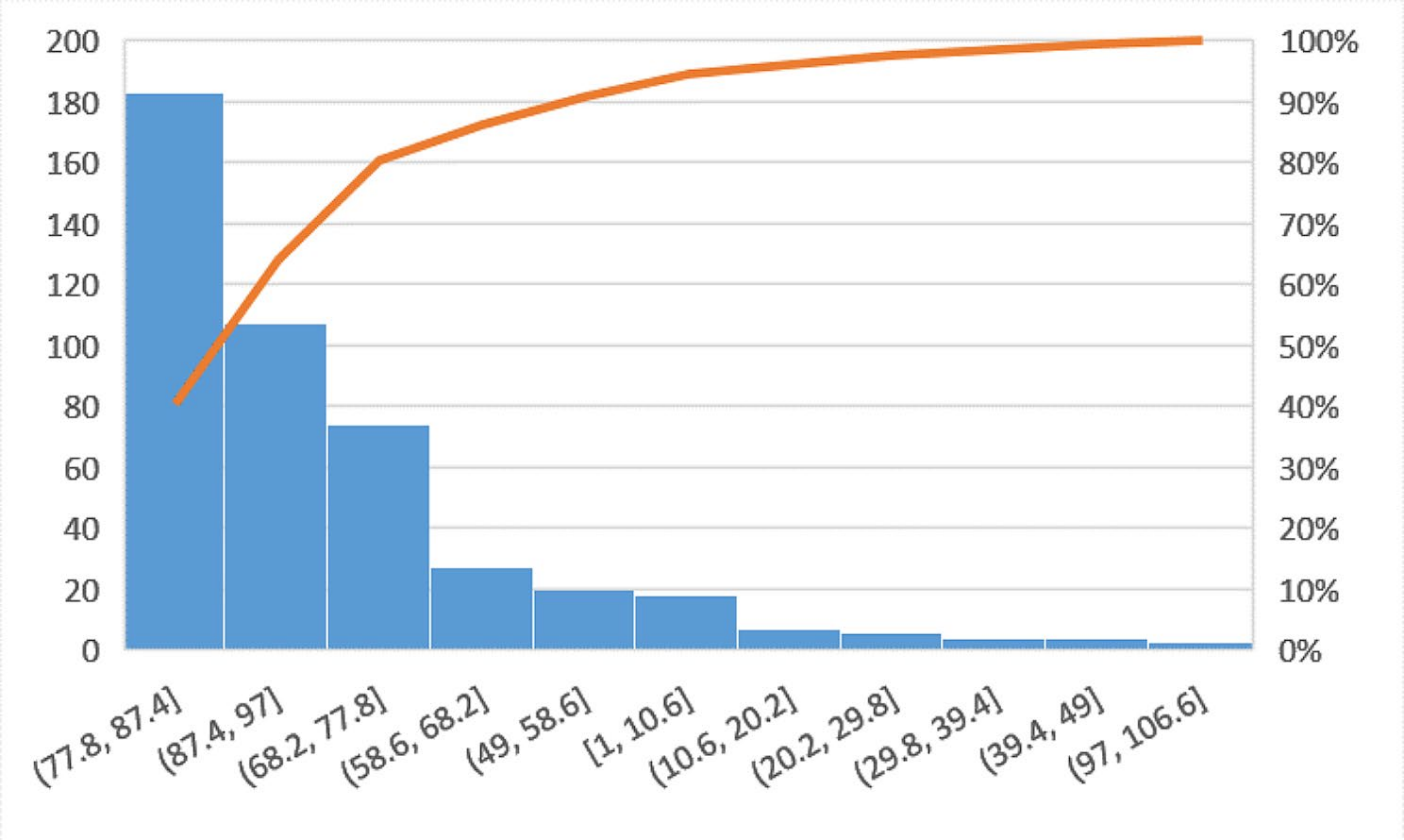
Distribution of Age in the dataset

As described in the Background, age is an important parameter for this specific type of fracture. For this reason, we decided to study through the Pareto Diagram (Fig. 3) the distribution of fractures by age in our dataset.

The analysis shows that 90% of the dataset is represented by patients older than 77 years, in line with what has been reported in the literature [1]. In order to obtain a sufficient number of patients to be able to train the ML algorithms, it was decided to extend the dataset to such a large number of years and to keep the information of the year of discharge among the independent variables of the model. In this way, it is possible to determine whether variations due to evolution, epidemiological and technological changes over time have influenced hospitalization.

### Pre-processing

After collecting the variables of interest, a preprocessing step needs to be performed before they are given as input to the classification algorithms.

For this study, we decided to use LOS-defined as the difference in days between the date of discharge and the date of admission-as the dependent variable, while all others listed above and appropriately coded were considered independent variables.

Using the list of secondary diagnoses in the discharge form, the comorbidity groups used here as independent variables in the model were derived using the national guidelines as a reference. No normalisation or standardisation techniques were adopted and, since this information flow is regulated by law, all the variables were characterised and, therefore, no cleaning techniques were used.

**Fig. 4:**
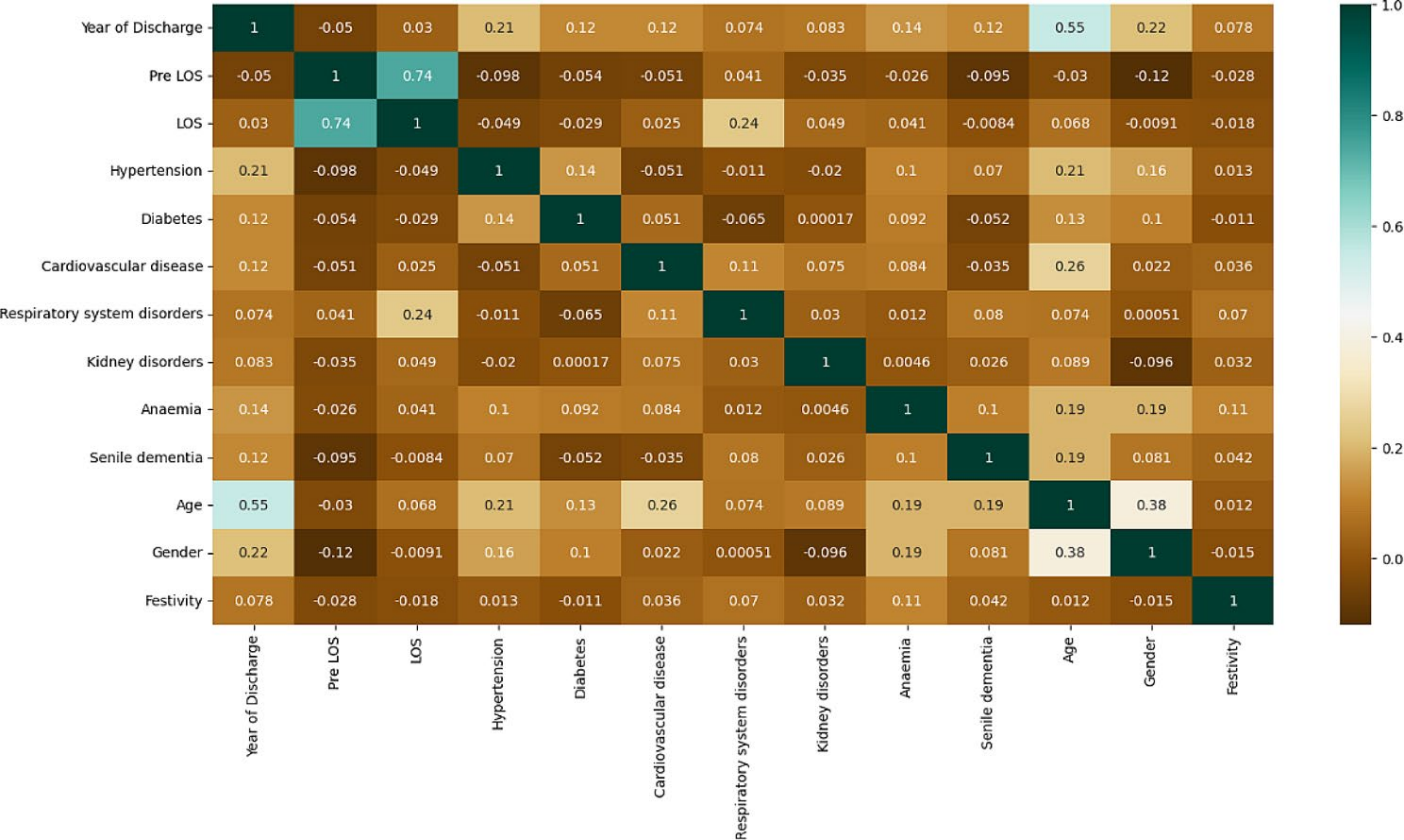
Pearson correlation results

Prior to the implementation of the algorithms, Pearson correlation was implemented. The results are shown in Fig. 4 using a colour map. Each cell takes on a colour ranging from brown (0) to dark green (1) depending on the correlation value returned, following the colour gradations reported by the coloured bar to the right of the chart.

In addition to the high correlation between LOS and Pre-Op LOS, there was a high positive correlation between LOS and respiratory system disorders and between age and year of discharge, gender and cardiovascular disease.

As defined, LOS is a continuous variable that cannot be studied by classification algorithms. Therefore, at this stage, arbitrary thresholds were defined for the division of the LOS. In particular, it was decided to use the week as the unit of measurement, as below:

0. LOS ≤ 7 days (*N* = 81);
1. 8 ≤ LOS ≤ 14 days (*N* = 318);
2. LOS ≥ 15 days (*N* = 54);

The division into classes was done not only to allow the study using classification algorithms, but also to prevent the effect of outliers. To avoid problems of imbalanced classification and thus low performance of the algorithms on the class with the least number of instances, it was decided to use oversampling. The choice of dividing the dataset by weeks strongly unbalanced the classes, in favour of the second one where 70% of the patients are included. The technique adopted was the Synthetic Minority Oversampling Technique (SMOTE), which allows increasing the number of data available based on the observations made on the dataset. Specifically, a random minority class observation is first selected and then the n (with n generally equal to 5) nearest neighbors for that example. At this point, one of the identified neighbors is selected and from it a new observation is created at a random point between the initial observation and its neighbor [38]. At the end of this stage, with *n* = 5 and not limiting the application to the minority class only, the dataset has a dimensionality of 954 patients. This process, as shown by Mohammed et al. [39] on health data or by Galassi et al. [40] in predicting the risk of a hip fracture, has a positive effect on the performance of the algorithms.

Finally, the dataset was divided into training (80% of the total) and test (20% of the total) datasets to calculate evaluation metrics. The split was performed in a random manner, which was used to test the algorithms. In the training phase, as will be seen in the next section, cross-validation techniques were also implemented to determine the best hyper-parameters.

### Machine learning algorithms

For LOS analysis in classes, it was decided to test and compare the results of the following six algorithms: Decision Tree (DT), Random Forest (RF), Gradient Boosted Trees (GBT), Support Vector Machine (SVM), Gaussian Naïve Bayes (GNB) and Multi-Layer Perceptron classifier (MLP). These algorithms were selected because they are proven to perform well in all application fields, including health care [41–43]. In addition to these, it was decided, to use Voting Classifier (VC) also. VC is an ML model that is trained using a set of different classification algorithms and determines the output based on a majority decision principle. Specifically, VC will contain the 6 algorithms listed above and observation x will belong to class y if at least 4 algorithms attribute it to that class (Hard type) or based on the average probability given to that class (Soft type). In this way, it is possible to exceed the performance of the single algorithm and obtain a better result.

As shown in Fig. 1, however, the implementation also includes a validation phase. Working on a given set of training/validation/tests on such a small dataset could lead to considerable loss of information. Therefore, Cross-Validation has been implemented. Molinaro et al. compared different validation methods on health datasets and showed that 10-fold cross-validation was among the techniques with the lowest bias [44]. This works by dividing the datatset into k random groups called folds, keeping one group out for testing and training the model on the remaining ones. This procedure will be repeated k times leaving one group out for testing each time. The final result is the average of the results obtained. Given the dimensionality of the dataset, it was decided to set k = 10 so as to obtain better results in a still acceptable processing time. Although there is no unambiguous k-value to which the best performance is associated, a sensible choice is k = 10 because the prediction error estimate is almost unbiased in a 10-fold cross-validation [45]. In addition to Cross-Validation, Grid Search for parameter selection was also implemented. This procedure makes it possible to automatically determine the best hyperparameters for a given model from intervals defined arbitrarily or based on literature / algorithm documentation by the user [43]. For each algorithm, the Grid Search CV is passed as input the grid of selected hyperparameters and the number of folds into which the dataset has been divided. At this point, the estimator API is used: after adaptation to a specific dataset, all possible combinations of parameters are evaluated until the best sequence is determined. For this work, the intervals used are shown in Table 1.

**Table 1:** Hyperparameter selection intervals

Algorithm	Hyperparameter
DT	‘criterion’: (‘gini’, ‘entropy’, ‘log_loss’) ‘max_depth’:range(3,20)
RF	‘n_estimators’: list(range(5, 20)), ‘max_depth’: list(range(2, 9)), ‘max_features’: (‘sqrt’, ‘log2’, None)
GBT	‘n_estimators’:[10, 100, 1000], ‘learning_rate’:[0.1, 1.0, 10, 100], ‘max_depth’:range(1,10)
SVM	‘kernel’:(‘linear’, ‘rbf’), ‘C’:[0.1, 1, 10, 100, 1000]
MLP	‘hidden_layer_sizes’: [(50,50,50), (50,100,50), (100,)], ‘activation’: [‘tanh’, ‘relu’], ‘solver’: [‘sgd’, ‘adam’], ‘alpha’: [0.0001, 0.05], ‘learning_rate’: [‘constant’,‘adaptive’]
GNB	‘var_smoothing’: np.logspace(0,-9, num = 100)
VC	‘Voting_technique’: (‘Hard’, ‘Soft’)

Once Cross-Validation was finished and the best hyperparameters were determined, it was possible to compare performance and determine the best algorithm. In addition to the metrics (Accuracy, Precision, Recall and F1-score), the performance of the best algorithm was also determined through the Receiver Operating Characteristic (ROC) curve for each of the classes. The sklearn library (sklearn.metrics.roc_curve) was always used for its implementation.

## Results

Before proceeding with the presentation of the results obtained, Table 2 shows for each algorithm the optimal combination of hyperparameters among the selected values. These make it possible to better fit the algorithm to the particular dataset under consideration and thus obtain the best results.

**Table 2:** Set of best hyperparameters for each algorithm

Algorithms	Best Hyperparameters
DT	{‘criterion’: ‘gini’, ‘max_depth’: 12}
RF	{‘max_depth’: 9, ‘n_estimators’: 20, ‘max_feature’: ‘sqrt’}
GBT	{‘learning_rate’: 1.0, ‘max_depth’: 3, ‘n_estimators’: 1000}
SVM	{‘C’: 100, ‘kernel’: ‘linear’}
MLP	{‘activation’: ‘tanh’, ‘alpha’: 0.05, ‘hidden_layer_sizes’: (100,,), ‘learning_rate’: ‘constant’, ‘solver’: ‘adam’}
GNB	{‘var_smoothing’: ‘0.000053’}
VC	{‘Voting_technique’:‘Hard’}

The algorithms thus defined were then tested. The evaluation metrics are shown in Table 3

**Table 3:** The performance parameters for the classification analysis

Class	Accuracy	Precision	Recall	F1-score
*DT*				
0	0.81	0.79	0.90	0.84
1		0.80	0.68	0.74
2		0.84	0.85	0.85
*RF*				
0	0.85	0.83	0.86	0.84
1		0.81	0.83	0.82
2		0.91	0.85	0.88
*GBT*				
0	0.83	0.81	0.83	0.82
1		0.81	0.79	0.80
2		0.86	0.87	0.86
*SVM*				
0	0.79	0.76	0.75	0.75
1		0.72	0.74	0.74
2		0.90	0.87	0.87
*MLP*				
0	0.81	0.80	0.87	0.83
1		0.74	0.80	0.77
2		0.92	0.74	0.82
*GNB*				
0	0.79	0.82	0.75	0.78
1		0.72	0.80	0.76
2		0.83	0.81	0.82

The best result was obtained by the RF algorithm with an accuracy of 85%, followed by GBT with 83% and DT and MLP with 81%. The confusion matrix proved that the model was capable of adequately identifying the class for the LOS (Table 4).

**Table 4:** Confusion matrix of the best ML algorithm

Real/ Predicted	0	1	2
0	54	9	0
1	6	55	5
2	5	4	53

The best results were obtained for class zero and class two with the least number of classification errors, worse result on the class one, where only 11 out of 66 cases are correctly classified.

Evaluating RF on the dataset without the SMOTE implementation resulted in a different set of better hyper-parameters {‘max_depth’: 5, ‘n_estimators’: 15, ‘max_feature’: ‘sqrt’}, a lower accuracy (0.85 VS 0.76) and a higher error rate in class 0 (14 out of 18 errors) and class 2 (6 out of 10 errors).

**Fig. 5:**
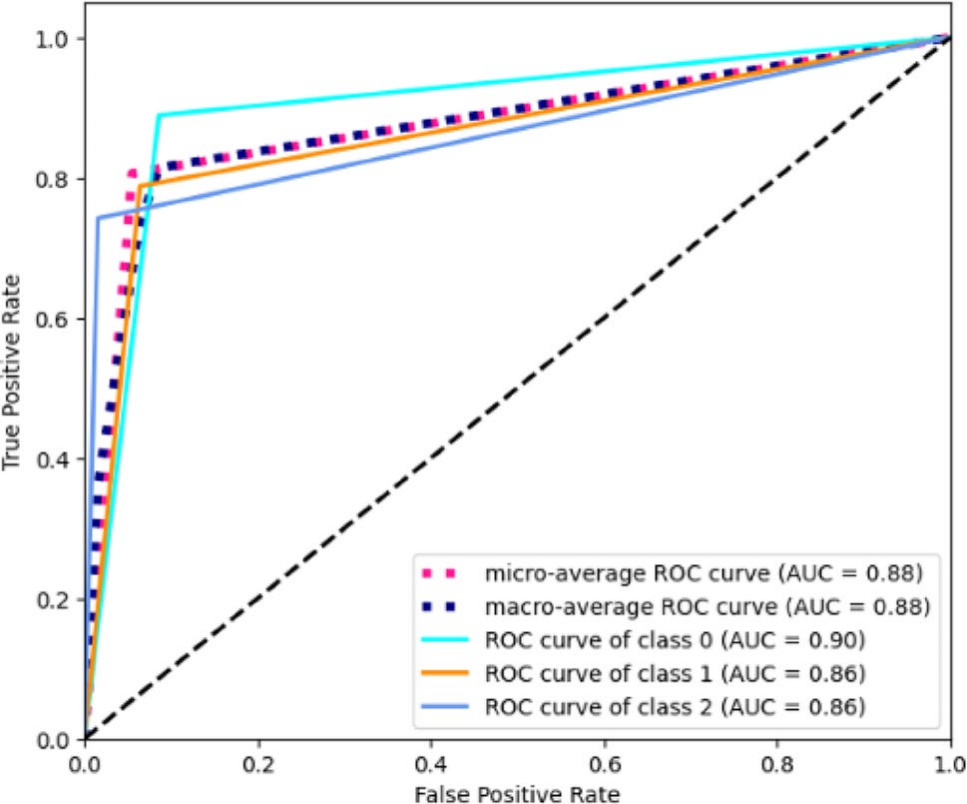
ROC curves

To complete the analysis of RF performance, ROC curves are shown below (Fig. 5).

Both the average ROC curves, micro and macro, reached an area of 0.88. As already shown by the confusion matrix, the best results are obtained for class 0 with an area of 0.90, followed by class one and two with 0.86.

**Fig. 6:**
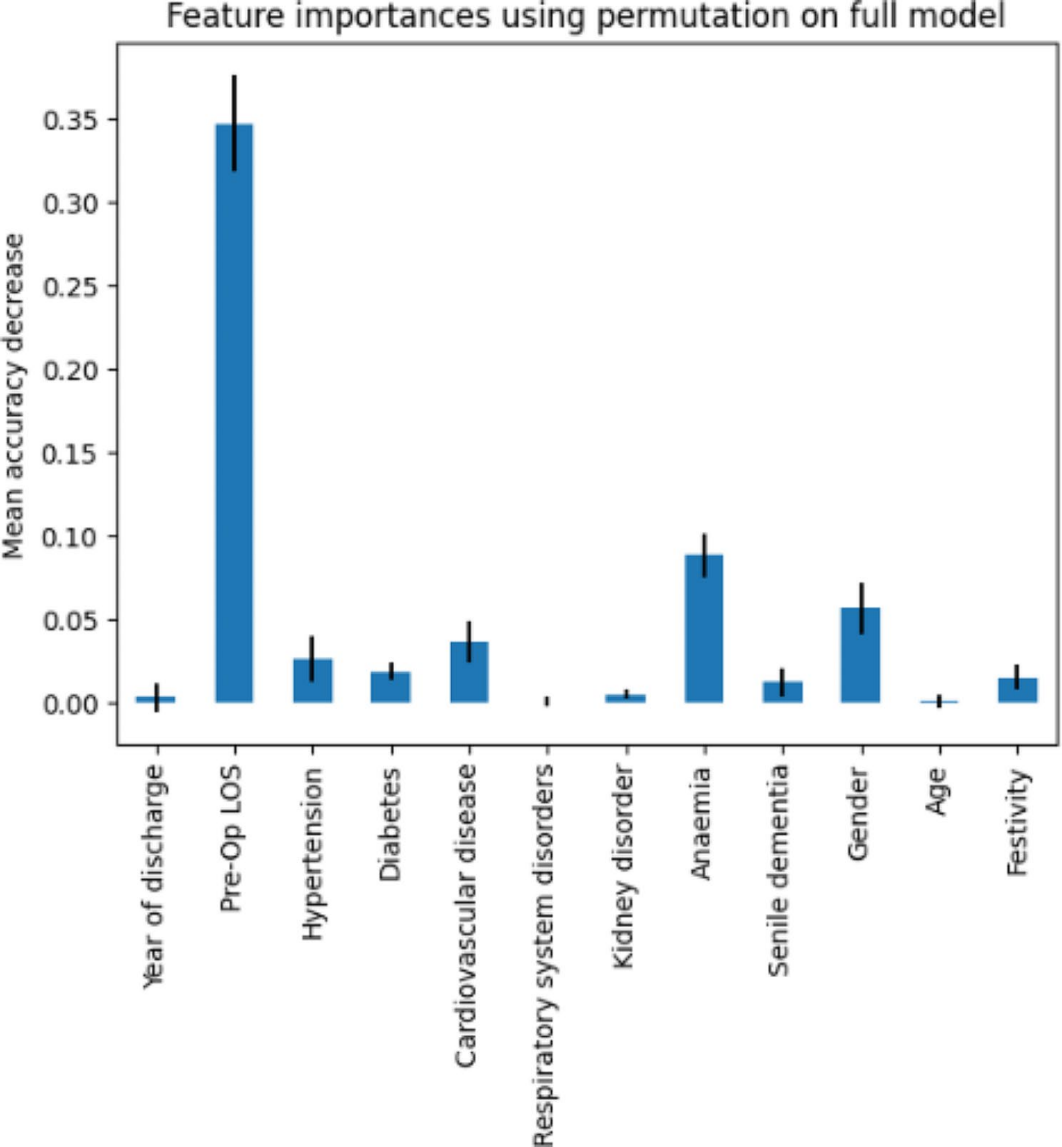
Histogram describing the feature importance for the DT model

Figure 6 shows the significance of the features of the RF classification analysis. Feature importance was obtained by calculating the accuracy of the RF model by replacing one of the independent variables with a corrupted version of it each time. The graph therefore shows the average reduction in accuracy obtained from this substitution.

The most important feature was preoperative-LOS followed by Anaemia and Gender. Some comorbidities such as respiratory system disorders and kidney disorder seemed to have no effect on hospitalization days.

Finally, VC was implemented. All previous models with optimised parameters were given input to VC. Separate Hard and Soft techniques were tested and from these, the one with the best result in terms of accuracy was chosen. Using a Hard voting technique, the best result was obtained in terms of accuracy of 0.87. Since the use of VC has a larger computational weight, dependent on the implementation of all the selected algorithms, it can be concluded that RF was the best.

## Discussion

The purpose of this work was to study the flow of patients accessing the hospital for a femoral neck fracture and needing surgery. The analysis was conducted at the “San Giovanni di Dio e Ruggi d’Aragona” University Hospital in Salerno, Italy, one of the prominent hospitals by catchment area in southern Italy. To do so, using hospital discharge records as an information source, different types of information (demographic, admission-related, and health-related) on patients admitted during the period 2005–2021 were collected. Classification algorithms were implemented using total LOS as the dependent variable of the models.

The results obtained showed generally good performance with an accuracy greater than 75.00%, in particular the best algorithm DT achieves a value exceeding 80%. The ROC curves helped us to demonstrate good results on the individual classes identified, with the area reaching a value of 0.90 on class 0 (patients with hospitalization within one week). Finally, the data analysis reveals several interesting tendencies in the factors that are strongly linked to LOS. Preoperative LOS, Admission Type and Discharge Mode accounted among the most influencing predictors of the overall LOS. As expected, age is also a determining factor showing that over 90% of the dataset consisted of patients over 45 years old.

As anticipated, the use of these techniques in the biomedical field is not new, but difficult to find, however, are applications to the femur, for which our work represents a novel element. Many studies discuss hip fracture, such as Zhao et al. [46] predicting postoperative delirium, Kremers et al. [47] highlighting how cost and LOS are directly related to patient obesity, while Elbattah et al. [48] use machine learning not only on LOS but also on discharge mode, which they state is also important for estimating the capacity of rehabilitation and long-term care facilities. From this, it is possible to justify the fact that in our study the type of admission significantly influences the LOS and thus the emergency access, which is the subject of the defined pathways within the hospital [9].

While these observations are in line with the available literature, the advantage of comparing different stratification models lies in the power of identifying those aspects that could be improved in order to design a more efficient process when taking in charge patients undergoing femur fracture surgery. Compared with the studies presented in the article, the analysis of risk factors is not limited simply to statistical analysis, but combines a correlation study with the implementation of ML algorithms. This system presented offers a tool that on the one hand makes it possible to characterize critical aspects of the pathway or determine focus areas (such as the presence of anemia) with which undesirable variability of hospitalization is associated, and on the other hand the possibility of prediction, a useful tool for bed management. In particular, the standardization of the preoperative procedures could have a considerable impact on the overall hospital stay by helping in reaching the main intervention in a shorter time. Here, therefore, an analysis tool is offered which goes further and allows a greater information content to be extracted from the data, although it does not integrate more precise data on the patient’s diagnosis and health status, as it does not have access to medical records. The latter does not allow a clear identification of patient classes, but on the other hand, the good performance obtained validates the tool even from administrative data alone. This study finds a number of demographics, pre-operative and post-operative factors that are substantially linked to LOS, as well as a formula for calculating predicted LOS in femur fractured patients. The multivariable analysis showed key drivers of LOS. This work could be valuable also for the selection and use of most appropriate algorithms to support the clinical decision-making and the value-based healthcare since it takes an important step toward risk stratifying patients according to their tendency to stay longer in the healthcare structure and, thereby, to consume more healthcare resources. This could both assist in patient management, through pre-operative consultations aimed at the management of the most critical comorbidities or the identification of specific pre-hospital and intra-hospital pathways and in bed planning since a range in which the patient could potentially be discharged is known. In order for the system to be usable, in addition to demographic information, comorbidities and possible complications must be accurately characterized. It should be noted that the solution offers a range estimate and not an exact value, precisely to take into account the intrinsic variability of each patient.

The limitations of the study are several. First, it is a single-center study that operates on a limited number of observations. This generates a selection bias, due to the hospital’s particular catchment area, which is also different from that which may be found in another Italian city. The information source did not allow a detailed characterization of the patient’s health status, as well as any complications that might occur during surgery. As anticipated, only administrative data were used and not the medical records. Access to this additional source of information would have made it possible to characterise the degree of severity of the identified comorbidities or to better clarify the complications that occurred. Further analysis techniques could be implemented or the understanding of the techniques used could be improved by means of explainability tools. In particular, the combined use of SMOTE, which generates dummy samples based on the observed dataset, and cross-validation could lead to the construction of biased models with over-optimistic error estimates [49]. Both this over-optimism associated with CV and overfitting due to oversampling will therefore need to be better investigated.

## Conclusion

In this study, conducted at the “San Giovanni di Dio e Ruggi d’Aragona” University Hospital in Salerno (Italy), 6 different classification algorithms were implemented to study the flow of patients arriving at the hospital due to a femoral neck fracture. This analysis made it possible to first identify the best model and then to identify the causes of prolonged hospitalization. The accuracy greater than 80% obtained pave the way for multiple future developments. Firstly, given the national focus on this disease, the aim will be to include data from different healthcare facilities in order to obtain generalizable results and not be limited to a single hospital. Access to medical records and the use of further data analysis techniques will make it possible to overcome the criticalities identified and increase the information pool and further improve patient management.

## Data Availability

The datasets generated and/or analyzed during the current study are not publicly available for privacy reasons but are available from the corresponding author on reasonable request.

## Abbreviations

LOS: Length Of Stay
DTAP: Diagnostic Therapeutic Assistance Pathway
ML: Machine Learning
DT: Decision Tree
SVM: Support Vector Machine
GBT: Gradient Boosted Trees
RF: Random Forest
MLP: Multi-layer Perceptron classifier
GNB: Gaussian Naive Bayes
VC: Voting Classifier
